# Innate and adaptive immunity in human epilepsies

**DOI:** 10.1111/epi.13784

**Published:** 2017-07-04

**Authors:** Jan Bauer, Albert J. Becker, Wassim Elyaman, Jukka Peltola, Stephan Rüegg, Maarten J. Titulaer, James A. Varley, Ettore Beghi

**Affiliations:** ^1^ Department of Neuroimmunology Center for Brain Research Medical University of Vienna Vienna Austria; ^2^ Section for Translational Epilepsy Research Department of Neuropathology University of Bonn ‐ Medical Center Bonn Germany; ^3^ Brigham and Women's Hospital and Harvard Medical School Boston Massachusetts U.S.A; ^4^ The Broad Institute Cambridge Massachusetts U.S.A; ^5^ Department Neurology Tampere University Hospital Tampere Finland; ^6^ Department Neurology University Hospital Basel Basel Switzerland; ^7^ Department Neurology Erasmus University Medical Center Rotterdam The Netherlands; ^8^ Nuffield Department Clinical Neurosciences John Radcliffe Hospital Oxford United Kingdom; ^9^ IRCCS‐Mario Negri Institute for Pharmacological Research Milano Italy

**Keywords:** Innate immunity, Adaptive immunity, Epilepsy, Autoantibodies, Encephalitides, Immunomodulatory drugs

## Abstract

Inflammatory mechanisms have been increasingly implicated in the origin of seizures and epilepsy. These mechanisms are involved in the genesis of encephalitides in which seizures are a common complaint. Experimental and clinical evidence suggests different inflammatory responses in the brains of patients with epilepsy depending on the etiology. In general, activation of both innate and adaptive immunity plays a role in refractory forms of epilepsy. Epilepsies in which seizures develop after infiltration of cells of the adaptive immune system in the central nervous system (CNS) include a broad range of epileptic disorders with different (known or unknown) etiologies. Infiltration of lymphocytes is observed in autoimmune epilepsies, especially the classical paraneoplastic encephalitides with antibodies against intracellular tumor antigens. The presence of lymphocytes in the CNS also has been found in focal cerebral dysplasia type 2 and in cortical tubers. Various autoantibodies have been shown to be associated with temporal lobe epilepsy (TLE) and hippocampal sclerosis of unknown etiology, which may be due to the presence of viral DNA. During the last decade, an increasing number of antineuronal autoantibodies directed against membranous epitopes have been discovered and are associated with various neurologic syndromes, including limbic encephalitis. A major challenge in epilepsy is to define biomarkers, which would allow the recognition of patient populations who might benefit from immune‐modulatory therapies. Some peripheral inflammatory markers appear to be differentially expressed in patients with medically controlled and medically refractory and, as such, could be used for diagnostic, prognostic, or therapeutic purposes. Establishing an autoimmune basis in patients with drug‐resistant epilepsy allows for efficacious and targeted immunotherapy. Although current immunotherapies can give great benefit to the correctly identified patient, there are limitations to their efficacy and they may have considerable side effects. Thus the identification of new immunomodulatory compounds remains of utmost importance.

**Key Points**

- Inflammatory mechanisms have been implicated in the origin of seizures in a number of encephalitides
- Activation of both innate and adaptive immunity seems to occur in refractory forms of epilepsy
- Antineuronal autoantibodies have been discovered and are associated with various neurologic syndromes, including limbic encephalitis
- Immunomodulation demonstrates incomplete efficacy with significant side effects
- The identification of new immunomodulatory compounds remains of utmost importance

Immune mechanisms have been discovered in several neurologic diseases, some of them associated with epilepsy.^1^ These mechanisms are not only present in epilepsies caused by infectious and central nervous system (CNS) inflammatory diseases,^2^, ^3^ but also in epileptic disorders not associated with a clear inflammatory pathophysiology.^4^ The exact role of the inflammatory phenomena (cause, effect, or both) is a matter of intense investigation. Rapid activation of proinflammatory cytokines and danger signals is observed after acute epileptogenic brain injuries or after single and recurrent seizures in both experimental and clinical settings. On the other hand, there is evidence of chronic overproduction of cytokines and other inflammatory mediators during epileptogenesis in animal models, implicating a neuromodulatory role of inflammation and its potential involvement in the generation of spontaneous seizures. The contributions summarized in this article investigate the role of immune mechanisms based on the results of recent and ongoing experiments to help improve our understanding of their role in epilepsy.

## Innate Immunity in Epileptic Disorders

(*Jan Bauer, Jukka Peltola*)

There is evidence supporting that several inflammatory mediators have a specific role in temporal lobe epilepsy (TLE) or in neocortical epilepsies associated with focal malformations. Rapid activation of proinflammatory cytokines, such as interleukin‐1β (IL‐1β), interleukin‐6 (IL‐6), and tumor necrosis factor‐α (TNF‐α), and danger signals, such as high mobility group box 1 (HMGB1)‐activating inflammasomes via Toll‐like receptors (TLRs), is observed after acute and chronic seizures in animal models of acquired epilepsies.^5^ Brain‐resident innate immune cells such as microglia as well as astrocytes are pivotal generators of this inflammatory response. There is also evidence of chronic overproduction of these molecules and other inflammatory mediators (e.g., cyclooxygenase‐2, prostaglandins, complement system components, and immunoproteasomes) in both glial cells and neurons, and in cellular components of the blood–brain barrier, in patients with TLE or malformations of cortical development, suggesting a neuromodulatory role of inflammation in epilepsy. Indeed, these inflammatory mediators were shown to play a role in the mechanisms of seizures and epileptogenesis in animal models.^6^, ^7^, ^8^, ^9^, ^10^ In particular, the presence of IL‐1β and HMGB1 has been shown in brains of patients with various epileptic disorders, such as TLE and focal cortical dysplasia (FCD).^6^, ^11^, ^12^ In addition to the local formation of seizure‐inducing molecules in the CNS, molecules such as IL‐1β, TNF‐α, and IL‐6 also might enter the brain from the blood. For this to happen, a breach in the blood–brain barrier (BBB) is necessary, and thus seizure induction by peripheral molecules only can play a role in disorders where the BBB is opened. In general, studies on human epileptic brain show the presence of ultrastructural changes and abnormal tight junctions of vasculature endothelial cells, indicating a breach in the BBB and the possibility that serum proteins may reach the CNS parenchyma.^13^ An example of this may be children with febrile seizures. Measurement of the above‐mentioned proconvulsant molecules in the plasma of these patients shows a clear increase.^14^ In addition, in an animal model of TLE, the BBB opening has been shown in the progression of the disease. The authors found albumin in the CNS following status epilepticus (SE) and a positive correlation between the extent of BBB opening and the number of seizures.^15^ On the other hand, it is unclear to what extent these molecules enter the CNS. Measurements of albumin and α2‐macroglobulin in the serum and cerebrospinal fluid (CSF) of children with febrile seizures show that most children have the same levels of albumin and α2‐macroglobulin in the CSF as controls but others clearly showed an increase of these molecules in the CSF.^16^

It is still unknown whether and in which cell types IL‐1β is expressed in forms of epilepsy characterized by extensive infiltration of adaptive immunity cells, such as Rasmussen encephalitis (RE). Bauer and colleagues therefore analyzed the presence of IL‐1β in RE using immune histochemical (IHC) studies, and they found selective presence of IL‐1β in microglial nodules in cortical gray matter as well as in subcortical white matter, in agreement with a recent report by a Ramashwamy et al.^17^ In the hippocampus, IL‐1β was not restricted to nodules, but was also present in surrounding microglial cells. This immunohistochemical data were supported by in situ hybridization (ISH) studies with a probe for IL‐1β messenger RNA (mRNA), which showed specific signals in microglial nodules. Astrocytes showed a weak immunostaining for IL‐1β but were negative for IL‐1β mRNA, raising the possibility that astrocytes may import the cytokine from the extracellular space, for example, via extracellular microvesicles. In addition to using IHC and ISH studies, these authors measured IL‐1β by western blot and enzyme‐linked immunosorbent assay (ELISA) in frozen sections from seven RE patients. Both analyses, however, did not show positive signals for IL‐1β, suggesting that the amount of recovered IL‐1β was under the detection level.

## Adaptive Immunity in Epileptic Disorders

(*Jan Bauer, Maarten J. Titulaer*)

### Infiltration of T lymphocytes

Experimental and clinical evidence suggests a different inflammatory response in the brain of patients with TLE, where the innate immune component appears to be prevalent, as compared to other refractory forms of epilepsy with prominent activation of both innate and adaptive immunity. Because innate and adaptive immunity reciprocally affect each other, it is important to recognize the presence and the composition of the inflammatory milieu in epileptic disorders.

**Figure 1:**
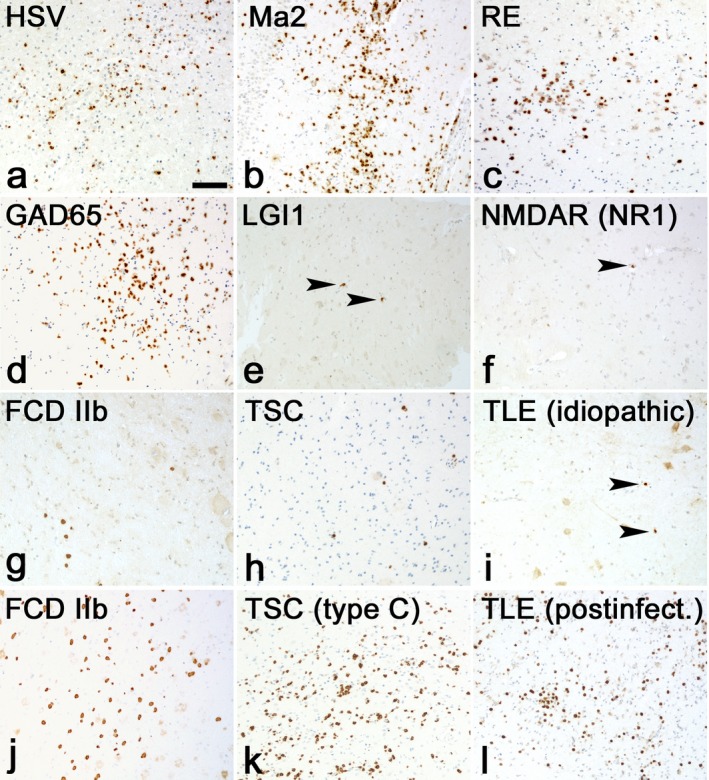
Comparative T‐cell inflammation in epilepsy. Staining for CD3^+^ T cells in (**A**, bar: 100 μm) HSV encephalitis, (**B**) anti‐Ma2 Ab paraneoplastic encephalitis, (**C**) Rasmussen encephalitis, (**D**) anti‐GAD65 Ab encephalitis, (**E**) anti‐LGI1 Ab Encephalitis, (**F**) anti‐NMDAR (NR1) Ab encephalitis. (**G**) FCD IIb with few T cells (**H**) TSC. This case contains only very few infiltrating lymphocytes (**I**) idiopathic TLE, (**J**) FCD IIb. Here the parenchyma reveals the presence of many T cells (**K**) TSC type C, with many T cells (**L**) postinfectious TLE. Numbers of inflammatory T cells are extremely high. All images have the same magnification. Single T lymphocytes are indicated by arrowheads.

Epilepsies in which seizures develop after infiltration of cells of the adaptive immune system in the CNS are an emerging group. It is increasingly recognized that this group consists of a broad range of epileptic disorders with different known and unknown etiologies (Fig.**** 1). Infiltration can be extremely abundant, in which case the epileptic disorder is designated as encephalitis. Examples of these include herpes simplex virus encephalitis^18^ and RE.^19^ A second group of epileptic disorders with dominant infiltration of lymphocytes are the autoimmune epilepsies (Fig.**** 1). These consist of classical paraneoplastic encephalitides with antibodies against intracellular tumor antigens such as Hu and Ma2, as well as paraneoplastic and nonparaneoplastic cases with antibodies against a large and still‐expanding range of intracellular and extracellular/membranous antigens, as discussed later. The number of infiltrating lymphocytes in paraneoplastic cases with antibodies against the various oncogenes is abundant. Moreover, most of these infiltrating CD3^+^ T lymphocytes are cytotoxic^20^, ^21^ and attack neurons.^22^, ^23^, ^24^ Whereas in paraneoplastic cases infiltration is severe, lymphocyte infiltration in other autoimmune epilepsies, in particular, those with antibodies against membrane antigens, is less abundant. Indeed, moderate infiltration of cells in cases of leucine‐rich glioma‐inactivated 1 (LGI1) encephalitis has been observed and lymphocytes are predominantly seen in the perivascular and interstitial spaces in cases of *N*‐methyl‐d‐aspartate receptor (NMDAR encephalitis)^24^, ^25^ (Fig.**** 1). Infiltration of lymphocytes in the CNS has also been found in a specific subgroup of FCD, that is, those composed of dysplastic neurons and balloon cells termed FCD type IIb by the International League against Epilepsy (ILAE) classification system.^12^ In most of the FCD cases, infiltration of T lymphocytes is relatively low. Bauer and colleagues confirmed that cases of FCD IIb contain moderate to high numbers of T cells and showed that some of these infiltrating lymphocytes were cytotoxic in nature. Finally, Aronica and colleagues recently reported that inflammatory infiltrates can also be present in a newly identified subgroup (type C) of cortical tubers.^26^ Bauer and colleagues support these findings, showing that CD3^+^CD8^+^ T lymphocytes are in close apposition to balloon cells, thus suggesting that cytotoxic T cells might specifically target these aberrant cell types.

Another large group of epileptic disorders in which infiltration of lymphocytes can be found are the cases of TLE with hippocampal sclerosis (HS) with unknown etiology. Bauer and colleagues presented data confirming that in some of these cases, infiltrating T cells are as numerous as in definitive limbic encephalitis (LE). Their preliminary results also revealed that especially one subgroup, that is, postencephalitic TLE cases, contained high numbers of infiltrating CD3^+^CD8^+^ T lymphocytes (Fig.**** 1) with a smaller subgroup of Granzyme‐B^+^ T cells.

## The Role of Viral DNA in Human TLE

(*Jan Bauer, Albert J. Becker*)

The reason for the high numbers of lymphocytes in postencephalitic TLE cases is unknown. Such inflammation might be due to the presence of viral DNA in the CNS. High human herpesvirus 6 (HHV‐6) DNA load has been detected in surgical tissue of patients with TLE, and viral DNA is most commonly present in patients with previous inflammatory brain diseases.^27^ However, detection rates have been found to vary considerably among studies. Becker and colleagues, in an initial limited series of 38 pharmacoresistant TLE patients versus 10 autopsy controls, detected HHV‐6 DNA in 55.6% of the TLE patients with a history of encephalitis, including mesial temporal sclerosis (MTS) and gliotic hippocampi without substantial neurodegeneration.^27^ They did not find HHV‐6 DNA in lesion‐associated TLE or nonlesional MTS with or without a history of complex febrile seizures (CFS). These data prompted them to carry out a subsequent large‐scale analysis of viral DNA/RNA spectrum in an extended series of TLE biopsies.^28^ In addition to all Herpesviridae, the group examined the presence of potentially relevant neurotropic RNA viruses. DNA and RNA were extracted from 346 fresh‐frozen epilepsy surgery tissue samples. Fresh‐frozen hippocampal tissue samples from 62 patients without chronic CNS disease served as controls. Real‐time polymerase chain reaction (PCR) and nested PCR were performed for Herpesviridae and RNA viruses, respectively. In addition, they analyzed the clinical records of the patients for the presence of earlier signs of inflammatory brain reactions. Their results revealed HHV‐6B DNA in 9.8% of the TLE patients and in 12.9% of the control samples. Intriguingly, however, the TLE samples were found to have a higher virus concentration. In patients with clinical signs of previous brain inflammation, HHV‐6B DNA was observed in 15.0%, whereas only 6.3% of the samples from patients without febrile seizures or meningoencephalitis were positive for HHV‐6B DNA. A meta‐analysis of the eight HHV‐6 PCR studies revealed similar results. To summarize, the biopsy‐based study of Becker and colleagues revealed no differences in frequency of HHV‐6B DNA detection between TLE patients and controls. However, the higher virus load in TLE patients and the increased detection rate of HHV‐6B DNA in patients with previous inflammatory brain reactions may be in line with a potential promoting role of HHV‐6 in TLE patients with a history of encephalitis. It is interesting to note that especially postencephalitic TLE cases revealed increased presence of cytotoxic lymphocytes, as presented by Bauer and colleagues. As such, new coordinated multicenter attempts should be helpful to shed more light on the role of HHV‐6 as well as other neurotropic viruses in the emergence of TLE. The abundance of viral nucleic acids in epileptic hippocampal tissue represents an aspect that requires particular attention by future studies.

## The Role of Immunologic Biomarkers in Refractory Epilepsy

(*Wassim Elyaman*)

Considerable progress has been made in identifying circulating epilepsy biomarkers in animal models of epilepsy.^29^ However, a major challenge is to define biomarkers in human epilepsy to allow the recognition of appropriate patient populations that might benefit from immunomodulatory therapies. Elyaman and colleagues proposed the hypothesis that peripheral inflammatory mediators are differentially expressed in patients with medically controlled and medically refractory epilepsy and, as such, could be used as a biomarker for diagnostic, prognostic, or therapeutic purposes. To characterize the regulation of circulating inflammatory mediators in epilepsy, Elyaman and colleagues assembled a multidisciplinary team that includes epileptologists, immunologists, epigeneticists, and computational biologists to generate comprehensive proteomic and transcriptomic data of peripheral (blood) and central (surgical tissues) adaptive and innate immune cells of a large pool of patients with epilepsy. Findings from these studies may give insight into the complex role of inflammation in the generation and exacerbation of epilepsy. In addition, it should yield new molecular targets for the design of antiepileptic drugs, which might not only inhibit the symptoms of this disorder, but also prevent disease pathogenesis.

## Antibodies and Epilepsy

(*Maarten J. Titulaer*)

In the past 30 years >30 antineuronal autoantibodies (ANABs) have been discovered.^30^ These ANABs are associated with various neurologic syndromes, which include LE, epilepsy, cerebellar degeneration, peripheral (poly)neuropathy, Lambert‐Eaton myasthenic syndrome (LEMS), opsoclonus‐myoclonus syndrome progressive encephalomyelitis with rigidity and myoclonus (PERM), and stiff‐person syndrome (SPS). At present, it is unclear how the formation of these antibodies is triggered. The induction of antibodies against the NMDAR has been associated with post‐herpes simplex virus encephalitis,^31^, ^32^, ^33^ but for other antibodies such an association is absent. The exact role of these antibodies in seizure induction remains the focus of research. These investigations have already revealed important mechanistic insight for a minority of antibodies.

In the nineties, voltage‐gated potassium channel (VGKC) antibodies were described in association with neuromyotonia.^34^ In the following years, these antibodies were found to be present in patients with LE and in Morvan's syndrome.^35^ More recently, it was shown that about half of these antibodies are directed against proteins complexed with the VGKC rather than the channel itself. Most patients have been found to have antibodies against LGI1.^36^, ^37^ A smaller group has antibodies against contactin‐associated protein‐like 2 (Caspr2), which is situated in the juxtaparanodal loop of myelin surrounding axons.^37^, ^38^ Only a minority of patients have antibodies against contactin‐2.^37^ Recently, a proportion of patients with positive VGKC‐complex antibodies but negative LGI1 and caspr2 antigen specific tests have been found to contain antibodies against the internal surface of the VGKC.^39^ The clinical significance of these particular antibodies is unclear, but currently there is no evidence for a pathogenic role for a positive VGKC test in the absence of LGI1 or Caspr2 antibodies.^40^ Therefore, the term VGKC is not useful and diseases should be referred to by their confirmed target antigen.

Mechanistically, anti‐LGI1 antibodies seem to affect the binding of LGI1 to disintegrin and metalloproteinase domain‐containing protein 22 (ADAM22), and thereby prevent proper signaling of the presynaptic VGKC and the postsynaptic α‐amino‐3‐hydroxy‐5‐methyl‐4‐isoxazolepropionic acid (AMPA) receptor.^41^ The mechanism of anti‐caspr2 antibodies has not been studied yet, but pathologic investigation of a caspr2 encephalitis brain showed immunoglobulin and complement deposition associated with neurodegeneration in the hippocampus.^42^ This antibody and complement deposition–associated neurodegeneration was also found in hippocampi of patients with anti‐LGI1 encephalitis, suggesting that, at least in these particular cases, the caspr2 and LGI1 antibodies can induce neurodegeneration via antibody‐dependent complement‐mediated cytotoxicity.^24^ All LGI1 and caspr2 sera contain (non–complement‐activating) IgG4 subclass antibodies.^43^, ^44^ In addition to IgG4, IgG1 or IgG2 subclass antibodies were found to some extent in 42% of LGI1 and 63% of caspr2 sera. These IgG1 and IgG2 antibodies are complement‐activating and therefore might be responsible for the hippocampal neurodegeneration described in some patients.^24^, ^42^

Because the antibodies against surface antigens have been discovered only recently, knowledge of the exact pathophysiologic mechanisms of such antibodies is still expanding. Anti‐NMDAR antibody was the first antibody to be discovered reacting to an extracellular antigen.^45^ Anti‐NMDAR encephalitis at present is also the most frequent autoimmune epilepsy. Most of the mechanistic insight for anti‐NMDAR antibodies has been described by Dalmau and colleagues.^46^, ^47^, ^48^ In vivo, the antibodies seem to affect the localization of NMDAR on the synapses of inhibitory neurons, by breaking the link with EphrinB2.^49^ This change in localization somehow leads to internalization of the NMDAR and a net decrease in NMDAR.^48^, ^50^ In vitro and in vivo studies in mice reveal that the decrease of NMDAR on the synapse is reversible after removal of antibodies.^51^ Furthermore, in vivo experiments suggest that an overload of EphrinB2 ligand can prevent the NMDAR internalization, and partially prevent the clinical phenotype. This could offer alternative symptomatic treatments in the future. Unlike in LGI1 and Caspr2 encephalitis, no major pathologic effect on individual receptor function or cytotoxic or complement‐mediated effects have been found.^24^, ^25^, ^52^ A question therefore remains why IgG complement–activating subclass antibodies from NMDAR encephalitis patients do not seem to induce complement‐mediated pathology.

## Clinical Aspects of Immunity in Epilepsy

(*Stephan Rüegg, Maarten J. Titulaer*)

From an epileptologic point of view, LE is the most important immune syndrome associated with the newly discovered ANABs. The main features of the syndrome are subacute onset of working memory deficits, altered mental status, confusion, delirium, or psychiatric symptoms. Furthermore, one can find progressive impairment of consciousness up to coma. For the possibility of an LE, at least one of the following observations should be present: de novo seizures or even status epilepticus, a new focal neurologic deficit, CSF pleocytosis, or MRI features suggestive of encephalitis. Excluding alternative causes for the patient's condition is also important. Swift recognition of the disorder is key because there are effective therapies and data increasingly suggest that early start of treatment is associated with better outcome.^30^, ^53^ Excellent reviews on this topic have been published recently.^54^, ^55^, ^56^, ^57^, ^58^

Another important antibody associated with epilepsy was the entity directed against the cytosolic enzyme glutamate decarboxylase (GAD). GAD catalyzes the conversion from glutamate to γ‐aminobutyric acid (GABA), and the presence of antibodies against GAD are associated with epilepsy,^59^ stiff person syndrome (SPS), and LE^60^, ^61^ (Table 1). Most of the early detected ANABs (detected before 2007) recognize intracellular epitopes and were associated with tumors causing paraneoplastic syndromes, whereas those ANABs identified more recently are less frequently paraneoplastic and are directed mainly toward antigens at the cell membrane. Anti‐GAD antibody encephalitis is an exception because the antibodies against intracellular antigens are induced in the absence of a tumor. A controversial transient extracellular availability at the synapse has been suggested to explain their pathogenicity.^62^ Since 2007, many new ANABs have been discovered (Table**** 2) and the clinical diversity of the ANAB‐mediated syndromes has largely expanded, maintaining the link with epilepsy in most of these ANABs. These aspects are discussed in brief below.

**Table 1:** Antibodies associated with epilepsy: state of art in 2010

Antibody	Epitope location	(Non‐)paraneoplastic	Refs
Anti‐Hu	Intracellular	Paraneoplastic	118, 119
Anti‐Ma1;Ma2/anti‐Ta	Intracellular	Paraneoplastic	120, 121
Anti‐amphiphysin	Membranous	Paraneoplastic	92
Anti‐Ri	Intracellular	Paraneoplastic	122
Anti‐AMPAR (GluR1/2)	Membranous	2/3 Paraneoplastic	73
Anti‐GABA_B_R	Membranous	1/2 Paraneoplastic	123
Anti‐GAD	Cytosolic	ca. 10% Paraneoplastic	59
Anti‐AMPAR (GluR3)	Membranous	Nonparaneoplastic	124
Anti‐NMDAR (NR1/2)	Membranous	1/3 Paraneoplastic	45
Anti‐LGI1	Membranous	10% Paraneoplastic	36
Anti‐Caspr2	Membranous	20% Paraneoplastic	37

**Table 2:** Antibodies associated with epilepsy discovered since 2010

Antibody	Epitope location	(Non)paraneoplastic	Refs
Anti‐mGluR5	Membranous	Paraneoplastic	79
Anti‐DPPX‐6	Membranous	Rarely paraneoplastic	82
Anti‐GABA_A_R	Membranous	Merely non‐paraneoplastic	88
Anti‐glycineR	Membranous	Non‐paraneoplastic	95, 125
Anti‐GABA_A_R	Membranous	Merely non‐paraneoplastic	90

### Anti‐NMDAR encephalitis

In 2013, Titulaer et al.^53^ presented a large cohort of 577 patients, including 212 children, with NMDAR encephalitis, and reported precise data on clinical features, treatment, and outcome. The majority (81%) had a good outcome (modified Rankin outcome scale 0–2), whereas 6% died. Prognostic factors predisposing to a good outcome in both adults and children were early onset of treatment and nonadmission to an intensive care unit. Relapse risk was 12% within the first 24 months, but relapses tended to be milder than the initial manifestation. The EEG phenomenon of “extreme delta brush” (extensive frontal beta activity superimposed on almost generalized rhythmic high‐amplitude delta activity) occurs in about 30% of severely affected intensive care unit (ICU)–bound patients and is thought to be specific for the disorder.^63^ Psychiatric symptoms are important in the early course of the disease, as 75% of adult patients are seen initially by a psychiatrist.^64^ These symptoms were the initial manifestation in 59% of the national French cohort (n = 111), with 40% experiencing hallucinations and 23% depression. A minority of these patients were drug resistant for antipsychotics or antidepressants. Referral to a neurologic (intensive care) unit was for many other causes like evolving seizures and status epilepticus and, in particular, for development of a neuroleptic malignant syndrome in 21 patients. Forty percent of the patients were first hospitalized in psychiatric institutions, however, more than half of these patients also had at least one neurologic sign and further 38% developed neurologic symptoms within days. These findings confirm previous studies of anti‐NMDAR encephalitis cases at psychiatric wards^65^ and ask for careful neurologic (re‐)examination of patients with new‐onset, relatively acute psychiatric illness.^66^ In young children, seizures and dyskinesias are the initial symptoms in half of the patients. In this group, psychiatric features as presenting symptoms, however, are less frequent (about one‐ third).^53^ The symptoms of the disorder evolve gradually and seem to resolve in reverse order of onset.^67^

### Anti‐LGI1 encephalitis

Shortly after the discovery of the LGI1‐associated LE, Irani et al.^68^ reported a unique epileptic syndrome of “faciobrachial dystonic seizures” (FBDS), with other groups reporting similar semiologies in the same context.^69^ These seizures are very short, lasting about 1–3 s, and consist of short dystonic stiffening of one arm and grimacing of the ipsilateral mimic musculature, or, less frequently, the leg. They may occur up to 50–100 times per day and, in 70% of cases, they precede the onset of LE by an average of 35 days. About 10% of patients do not develop LE. Due to their unusual and characteristic semiology, FBDS may be missed or misinterpreted as “psychogenic” or “tics.” Electroencephalography (EEG) rarely shows epileptic abnormalities.^68^ LGI1 encephalitis is the second most frequent autoimmune encephalitis, with an incidence of slightly less than one per million.^40^ It is infrequently (10–15%) associated with tumors (thymoma, lung cancer), and is slightly more prevalent in men and in patients >50 years. Seizures are either focal in the early stages (FBDS or stereotypic focal seizures with dyscognitive or autonomic features)^68^ or generalized tonic–clonic, a late symptom as part of an LE. With the onset of LE, memory loss, confusion, and personality changes are frequent.^70^ Mild to moderate hyponatremia (115–130, most over 125 mmol/L) is present at onset in two thirds of patients. The outcomes can be serious, as 65% of patients have persistent mild to severe cognitive deficits.^40^, ^71^ In addition, 13% of patients develop a cognitive encephalopathy without seizures.^43^ After adequate treatment, anti‐LGI1 encephalitis evolves into chronic epilepsy in one in seven patients.

### Anti‐Caspr2 encephalitis

More rare than LGI1 encephalitis, anti‐Caspr2 encephalitis has been reported in about 100 patients to date. Every fifth patient has paraneoplastic disease (lung, colon cancer, thymoma). There is strong preponderance of male patients (60–95% in different series), mainly older than age 60. Patients can present with LE, ataxia, peripheral nervous system involvement (neuromuscular hyperexcitability, including neuromyotonia, or pain) or a combination of peripheral and central symptoms and signs, such as Morvan's syndrome.^44^, ^71^, ^72^ Seizures occur in 50–90% of patients.^44^

### Anti‐AMPAR encephalitis

After a first series of 10 patients,^73^ there are now >50 patients reported with AMPA encephalitis, which has a paraneoplastic (thymoma, lung cancer) background in two thirds of these patients.^73^, ^74^, ^75^ There is female preponderance and patients tend to be older than 40 years of age. The following four types have been reported: (1) a confusional type with predominant agitation up to psychosis; (2) a mainly amnestic form; (3) an epileptic form with generalized and more rare focal seizures, with impairment of consciousness; and (4) a fulminant type with a severe course. Seizures occur in 20–40% of patients only, and patients tend to relapse although treatment response is good even in these recurrent episodes.^72^, ^74^

### Anti‐GABA_B_R encephalitis

This form of encephalitis is increasingly recognized and among the most prevalent after NMDAR and LGI1 encephalitis. More than half of cases are paraneoplastic, mainly associated with small cell lung cancer. Of interest, anti‐GABA_B_ receptor antibodies are absent in large cohorts of patients with small cell lung cancer, indicating that the presence of the antibodies can exert their pathogenic effect in the absence of a tumor. There is preponderance of male patients older than 40 years who present with seizures (partial complex, secondarily generalized seizures, or frequently refractory status epilepticus [SE]). In addition, patients have memory loss, confusion, and severe insomnia. Rare symptoms include ataxia and opsoclonus/myoclonus syndrome.^76^, ^77^, ^78^

### Anti‐mGluR5 encephalitis

Encephalitis mediated by antibodies against the metabotropic glutamate receptor subtype 5 (mGluR5) is always paraneoplastic and associated with Hodgkin's lymphoma.^79^ The signs of this extremely rare condition (only seven cases reported) include marked and subacute memory loss, depression, delusions, hallucinations, and psychosis together with tumor symptoms of cachexia, fever, and night sweats. The combination of encephalitis, depression, delusion, and cachexia led to the term “Ophelia syndrome,” coined by the author of the first case (his own daughter).^80^ Antibodies against the mGluR1 receptor cause a completely different neurologic, cerebellar (ataxic) syndrome despite large homology of the mGluR1 receptor with the mGLuR5 receptor.^79^, ^81^

### Anti‐dipeptidyl‐peptidase‐like protein‐6 (DPPX) encephalitis

This is a rare syndrome where antibodies against the epitope DPPX of the voltage‐gated potassium channel Kv4.2 cause classical symptoms of LE with confusion, memory loss, psychosis, or depression, but additionally brainstem signs (eye movement disturbances, ataxia, dysarthria, dysphagia, and respiratory failure), sleep difficulties, and myoclonus. As a unique clinical hallmark, profuse and difficult to treat, noninfectious diarrhea precedes LE weeks to months in two thirds of cases.^82^, ^83^, ^84^ This may result from the expression of the epitope in both intestinal cells and neurons. Seizures are rarely observed (about 15%). The antibodies have also been found in cases of progressive encephalomyelitis with rigidity and myoclonus (PERM).^85^

### Anti‐GABA_A(β3/γ2)_R‐encephalitis

LE associated with antibodies against GABA_A_ receptor subunits β3 and γ2 is rare and, in two thirds of cases, affects men between 3 and 74 years. The reason for this gender disparity is unknown but interesting, since male predominance in autoimmune disorders is rare. One might speculate that female sex hormones, such as allopregnanolone, or oral contraceptives may consolidate γ2‐subunits^86^ and be protective against seizures and SE. GABA_A_ receptors, especially the extrasynaptic, can be modulated by neurosteroids with sex‐hormone–like structure.^87^ About 20% of patients have cancer. Main symptoms are the ones typical for LE, but 50–70% of patients have severe seizures, up to almost intractable SE. In addition, some patients also have SPS or opsoclonus/myoclonus syndrome. Response of seizures and SE to immunosuppression is remarkable, whereas classical antiseizure drugs are ineffective against epileptic activity.^88^, ^89^

### Anti‐GABA_A(α1/γ2)_ R encephalitis

A second type of anti‐GABA_A_ receptor antibody has been reported recently. These antibodies were discovered in a large cohort of patients tested for autoimmune CNS disease. The antibodies were directed against the receptor subtypes α1 and γ2, and high titers are associated with both seizures and memory impairment in 47% of patients, in 33% with hallucinations, and 20% with anxiety.^90^ A clear encephalitic syndrome could not be identified and important clinical information on these patients (titers in CSF, course of disease, effect of treatment, etc.) were lacking; thus these antibodies may not cause LE sensu stricto.^91^

### Anti‐amphiphysin encephalitis

Anti‐amphiphysin encephalitis has been very rarely reported. About 40% of cases were paraneoplastic and mainly caused by small lung cell and breast cancer. Men were more frequently affected, and mean age of onset is 54 years. The classical form includes memory loss, cognitive decline, mood changes, depression, and sometimes hallucinations up to a frank psychosis. Seizures occur in about 40% of patients (generalized > focal with impairment of consciousness). Some patients also experience brainstem signs, like vertigo, cranial nerve palsies, and ataxia.^92^, ^93^

### Anti‐glycineR encephalitis

Although about 3% of two large cohorts of adult patients with newly diagnosed and established epilepsy harbored antibodies against glycine receptors,^94^ only three young boys younger than age 6 years have been reported to have (sub‐)acute encephalitis, focal seizures, and even SE associated with these antibodies.^95^, ^96^, ^97^ The anti‐glycine receptor antibodies are normally linked to SPS and PERM.^98^

In summary, the incidence of all types of encephalitis is estimated at 1:15,000^99^ and the LE caused by ANABs is probably around 1:100,000, although exact epidemiologic data are lacking. The incidence of seizures in these cases strongly varies according to the specific type of ANABs (Table 3). Conversely, ANABs are found in about 1–3% of patients with newly established epilepsy, but in a higher proportion (10–15%) in pharmacoresistant patients.^94^, ^100^, ^101^ This remains an important area of uncertainty because the significance of the presence of ANABs in patients with epilepsy alone is still poorly understood, as is the value of antibody testing in high‐throughput cohorts, without further exploration of the significance of these positive results. Are they merely bystander or a reliable surrogate marker for pharmacoresistance when these patients were treated only with antiseizure drugs but not immunomodulating agents? Such treatments yielded remarkable therapeutic effects in one study of highly selected patients, but prospective randomized controlled studies are still lacking.^102^, ^103^

**Table 3:** Frequency of seizures during/after limbic encephalitis (LE)

Antineuronal antibody	Frequency of seizures	Incidence of LE
Anti‐GABA_B_R‐IgG_1_	90%	Low to medium
Anti‐GAD‐IgG	25–100%^a^	High
Anti‐Hu‐IgG	60–100%	Low to medium
Anti‐GABA_A_R‐IgG_1>3_	50–100%	Medium
Anti‐LGI1‐IgG_4>2>1_	90%	Medium to high
Anti‐NMDA1/2R‐IgG_1_	70%	High
Anti‐GABA_A(b3/g2)_R‐IgG_1>3_	47%	Medium
Anti‐Caspr2‐IgG_4>1_	20–65%	Low to medium
Anti‐Ma1/2‐IgG	30–40%	Low to medium
Anti‐AMPAR‐IgG_1_	33%	Low to medium
Anti‐DPPX‐IgG	15%	Low
Anti‐mGluR5‐IgG	20%	Very low
Anti‐amphiphysin‐IgG	10–20%	Low
Anti‐CV2/CRMP5‐IgG	Rare	Low to medium
Anti‐glycineR‐IgG	Rare	Low

a Depending on the definition of acute immune LE.

## Treatments in Autoimmune Encephalitis

(*James A. Vardley*)

The first‐line treatment of autoimmune encephalitis combines steroids with either plasma exchange or intravenous immunoglobulin (IVIG) or, occasionally, both. Second‐line treatments are with cyclophosphamide and rituximab.^53^ The best evidence for treatment of autoimmune encephalitis is presented in NMDAR‐Ab encephalitis, as this is the most common.^53^ Around 50% of NMDAR‐Ab patients respond to first‐line treatment with a “good outcome” of an modified Rankin scale (mRS) of 0–2 at 24‐month follow‐up. In the remaining 50%, a good outcome can be achieved by second‐line treatment. There are nuances in this data, as only 50% of patients studied retrospectively had follow‐up data at 24 months. Second‐line treatment also halved the relapse incidence in 25% of patients, and early treatment was associated with a higher chance of a better outcome, something that has been mirrored in a small prospective cohort in LGI‐Ab encephalitis.^104^ Evidence for rituximab is equivocal in LGI‐Ab encephalitis but these data are on a small sample size.^105^

In other antibody encephalitides, because of their rarity, there is a less clear idea of treatment responses. In general, around 70% of patients respond to treatment. Here, factors associated with a poor outcome include coexistent tumor, delay to treatment, and poor initial functional status. In addition, the higher mortality seen in cases associated with antibodies such as GABA_A_, GABA_B_, and AMPA‐receptor antibodies can be explained by the more frequent occurrence with tumors.^106^

## Next‐Generation Targeted Immunomodulatory Therapies

(*James A. Vardley*)

More specific targeted therapies are an attractive prospect, as the broad immunosuppressive therapies have a wide range of side effects that contribute significantly to morbidity. Efforts have been made using the following medications or interventions.

### Immunoabsorption

Plasma exchange is well established as a treatment option, but a trial of immunoabsorption (IA) was recently reported.^107^ Eighty‐six percent of patients with ANABs improved, whereas patients with intracellularly targeted antibodies antigens did not. Antibody clearance was also interesting, with titers decreasing by 97% (serum) and 64% (CSF) 4 days after IA and falling further to 98% (serum) and 88% (CSF) at 4 weeks, showing a prolonged treatment effect. It is worth noting that this is higher than reported in plasma exchange series^108^; however, those prior studies were published 20 years ago and recruited patients with intracellular antigens. The precise clinical effect of IA is confounded by the coadministration of immunotherapy in this study, but it does appear to be an effective treatment alongside standard immunotherapy and spares the use of precious transfusion products required with plasma exchange and avoids the associated side effects.

### Tocilizumab

B cells are thought to be crucial to the ongoing immune response in patients with ANABs. IL‐6 is a cytokine thought to be a key player in B‐cell maturation and terminal differentiation into plasma cells, which secrete copious antibody.^109^ A study of neuromyelitis optica (NMO) with aquaporin‐4 antibody–positive patients identified IL‐6 as a key cytokine for B‐cell maturation and disease‐specific antibody production.^110^ Preliminary data using Tocilizumab, an anti‐IL‐6 monoclonal antibody, in NMO and autoimmune encephalitis is encouraging, and more work must be done to assess the efficacy of this as a standalone second‐line therapy.^111^

### Low‐dose IL‐2

A Korean group has treated patients with tocilizumab in combination with low‐dose IL‐2 to stimulate T regulatory cells, which is known to reduce T‐cell activation and attenuate B‐cell maturation in germinal centers, with encouraging results.^112^ A caveat is that it is impossible to untangle the effect of individual treatments when multiple ones are given contemporaneously. This study is further hampered by small sample size and the absence of confirmation of neuronal surface antibodies in some of the patients.

### Bortezomib

Plasma cells are key components of the humoral immune response. Bortezomib was originally designed as a treatment for myeloma and functions by inhibiting proteasome function in cells.^113^ This is thought to affect the immune system in a variety of ways, but plasma cells, due to their protein synthesis, are strongly dependent on proteasomal function. Bortezomib, therefore, is hypothesized to cause an accumulation of proapoptotic factors and targeted cell death, especially in plasma cells. This treatment has been trialed in NMDAR‐Ab encephalitis in two refractory patients and demonstrated promising initial results.^107^ Further studies with carefully selected patients are needed.

Immunomodulatory treatments are not without adverse side effects. Adverse effects of immunoabsorption include colonization of catheter tip with coagulase‐negative staphylococci and venous air embolism.^107^ Upper respiratory tract infection and pharyngitis or nasopharyngitis can be frequently observed in patients receiving tacilizumab.^114^ In these patients, clinical laboratory abnormalities include neutropenia and elevated aminotransferase levels. One of the most common side effects of bortezomib is peripheral neuropathy that is predominantly sensory with burning paresthesia, hyperesthesia–hypoesthesia, neuropathic pain, and weakness.^115^

## Conclusions and Future Directions

Anti‐inflammatory drugs have been reported to control seizures in drug‐resistant epilepsy and in selected epilepsy syndromes even in the absence of a clear inflammatory basis. An autoimmune etiology is increasingly identified among patients with epilepsy in whom no unequivocal causes are detected with the present diagnostic aids. In addition, seizures can initiate brain inflammation in glial cells and promote BBB disruption independent of leukocytes or blood‐borne inflammatory molecules.^116^ However, with one exception,^117^ the present evidence of seizure reduction by anti‐inflammatory drugs in humans relies mostly on case reports or small series. In these cases, a chance association between the disease and biological markers of altered immunity is still possible. We cannot even confirm that drug resistance has an autoimmune basis in these patients because a more rigorous investigation of immune mechanisms is typically performed in patients with the most severe disease varieties. Research in the field of autoimmune epilepsies is at a fast pace, with multiple new autoantibodies discovered every year. Numerous questions must be answered. These can be divided in those regarding basic mechanisms and those regarding clinical evaluation and therapy. For instance, at present it is completely unclear why antibodies such as anti‐LGI mostly target the limbic regions but almost no pathologic or clinical changes are found in regions that also have high concentrations of the target antigen such as the cerebellum. Some of the more basic questions that therefore need to be addressed in the future are the concept of antibody formation, the mechanisms by which the antibody gains access to the CNS, and the reason that the BBB (for instance in anti‐LGI encephalitis) seems to be breached mostly in limbic structures. Furthermore, the association of herpes simplex virus with anti‐NMDAR antibodies suggests that viral infection, at least in some patients with anti‐NMDAR encephalitis, might facilitate a break in tolerance giving rise to disease. It is still unclear whether other antibody‐associated limbic epilepsies are also secondary to a primary (viral) infection.

From a clinical point of view the search for further antibodies is required because this may decrease the number of patients with clinical, MRI, and CSF findings of LE but no identifiable ANABs. At the same time, it is important that awareness of characteristic clinical syndromes is improved to allow for early identification and treatment, given the beneficial effect of prompt treatment on outcome. In addition, the question should be asked whether it is useful that patients with pharmacoresistant epilepsy are mandatorily screened for ANABs and what antibody results warrant which types of treatment. Establishing an autoimmune basis in patients with drug‐resistant epilepsy may help investigate the efficacy of drugs active on the immune system.

The first‐line treatment of autoimmune encephalitis combines steroids with plasma exchange and/or IVIG. However, these treatments have been assessed only in observational studies. To improve therapeutic interventions, it is important to collect treatment data from prospective controlled randomized trials; however, this is problematic given the rarity of these conditions. Finally, immunomodulation demonstrates impressive but incomplete efficacy with significant side‐effect profiles as shown earlier. For these reasons, the identification of new immunomodulatory compounds with better tolerability and safety profile remains of utmost importance.

## Disclosure

We have no conflicts of interest with regard to this manuscript. We confirm that we have read the Journal's position on issues involved in ethical publication and affirm that this report is consistent with those guidelines.
